# Formation of Molybdenum Blue Nanoparticles in the Organic Reducing Area

**DOI:** 10.3390/molecules26154438

**Published:** 2021-07-23

**Authors:** Maria Myachina, Natalia Gavrilova, Victor Nazarov

**Affiliations:** Department of Colloid Chemistry, D. Mendeleev University of Chemical Technology of Russia, Miusskaya sq., 9, 125047 Moscow, Russia; ngavrilova@muctr.ru (N.G.); nazarov@muctr.ru (V.N.)

**Keywords:** molybdenum blue, self-assembly, polyoxometalate, molybdenum oxide, sol-gel method, particle formation kinetics

## Abstract

Molybdenum blue dispersions were synthesized by reducing an acidic molybdate solution with glucose, hydroquinone and ascorbic acid. The influence of the H/Mo molar ratio on the rate of formation of molybdenum particles was established. For each reducing agent, were determined the rate constant and the order of the particle formation and were established the conditions for the formation of aggregative stable dispersion with the maximum concentration of particles. The dispersed phase is represented by toroidal molybdenum oxide nanoclusters, which was confirmed by the results of UV/Vis, FTIR, XPS spectroscopy and DLS.

## 1. Introduction

Molybdenum-oxygen nanoclusters represent a large class of polyoxometalates (POM) [1,2,3,4,5]. The modern chemistry of polyoxometalates is very extensive and includes clusters of various chemical composition, size and shape [5,6,7,8,9,10,11,12]. Usually disperse phase of molybdenum blue dispersions is presented by giant clusters, which are formed as a result of self-assembly from the original building blocks, which are Mo_1_, Mo_2_, Mo_8_, etc. [13,14,15,16].

POMs, in particular molybdenum oxide nanoclusters, are characterized by a small particle size of the order of 3 nm, are formed spontaneously under certain conditions and have an amazing resistance to the action of electrolytes. By their behavior, dispersions of molybdenum blue are close to micellar solutions of surfactants, classical representatives of associated colloids [8,10,17,18,19]. Polyoxometalates exhibit remarkable physicochemical properties, structural versality and high reactivity. These systems are considered as the most promising precursors for production of hybrid materials, drug delivery systems, nanoreactors and catalytic materials [20,21,22,23].

It should be noted that in the case of dispersions of molybdenum oxide nanoclusters, lyophilization of the surface is provided by the formation of a hydrated shelf on the nanocluster surface. However, this factor is not sufficient to ensure the aggregative stability of this type of dispersed systems. That is, unlike associated colloids, molybdenum blue dispersions still require additional stabilization. Due to the features of the synthesis of polyoxometalate complexes, a reducing agent, a necessary component in the process of self-assembly of clusters, can act as a stabilizer. This role can also be performed by the oxidation products of some of the organic reducing agents. Moreover, if organic compounds are chosen as a reducing agent, then it is possible to immediately solve not only the problem of the formation of particles (clusters), but their further stabilization also [24,25,26,27]. Next, the presence of organic compounds in the synthesized dispersion allows to prepare carbides without the introduction of additional components, for example.

Besides it, the reducing agent has to match some other conditions, among which it is the redox potential sufficient for the partial reduction of Mo^+6^ compounds, high solubility and further chemical stability of oxidized products in an aqueous solution. The following organic compounds fulfill these requirements: glucose, hydroquinone and ascorbic acid. In our previous works, it was shown that these reducing agents make it possible to synthesize stable dispersions of molybdenum blue [28,29,30,31,32]. Previous studies were dedicated to the search of the optimal synthesis conditions, but not to the rigorous investigation of the self-assembly of molybdenum blue nanoparticles and the role of the reducing agent in this process. 

However, the difference in the structure of these organic compounds should affect the process of self-organization of molybdenum blue nanoparticles and the further behavior of the dispersed system in time. Taking into account the previously obtained data, we assumed that hydroquinone acts only as a redox agent, glucose and ascorbic acid, in addition to the reducing function, play the role of a stabilizer.

A study of the processes leading to the formation of particles in stable colloidal systems would lead to a more complete understanding of the synthesis of molybdenum blue as colloidal systems. The scientific novelty of this work is in the results of the comparative analysis of the kinetic study of the particle formation and the behavior in time of dispersions of molybdenum blue, synthesized using organic reducing agents of various nature. Such an analysis will be carried out for the first time and will make it possible to establish the factors of aggregation stability of systems unique for colloidal chemistry—dispersions of molybdenum blue.

The aim of this work was to investigate the properties of molybdenum blue dispersions and to estimate the role of each organic reducing agent in the process of self-assembly of nanoparticles. 

## 2. Results

### 2.1. Kinetic Study of Nanoparticles Self-Assembly

It is known that the formation of clusters of molybdenum blue occurs as a result of self-organization (self-assembly) of molybdenum complexes [33]. For the process of self-organization, the presence of certain complexes of Mo^+5^ and Mo^+6^ is required. For the obtaining of Mo^+5^ complexes it is necessary to carry out a partial reduction of the molybdate ions in solutions [34]. In this case the self-organization process is possible only at a certain pH of the dispersion medium (pH < 2) [35]. It is in such a medium that polycondensation of molybdate ions is observed with their subsequent organization into large molybdenum oxide clusters (particles of molybdenum blue).

Thus, to obtain dispersions of molybdenum clusters (herein after referred to as molybdenum blue), it is necessary to determine the optimal molar ratios of the reagents: reducing agent/Mo (R/Mo), acid/Mo (H/Mo) and, also, to establish the pH value at which stable molybdenum blue hydrosols are formed. In this work, glucose, hydroquinone and ascorbic acid were used as the reducing agent. 

Earlier research [17,18,19] found that the formation of aggregative stable molybdenum blue occurs at a certain (R/Mo) ratio and for each reducing agent there is an optimal ratio. For example, when using glucose, the required ratio (R/Mo) is 7/1, hydroquinone 4/1 and 1/1 for ascorbic acid. Under these conditions the maximum number of molybdenum oxide clusters is formed. These parameters were used in this work for the synthesis of stable dispersions

**Figure 1:**
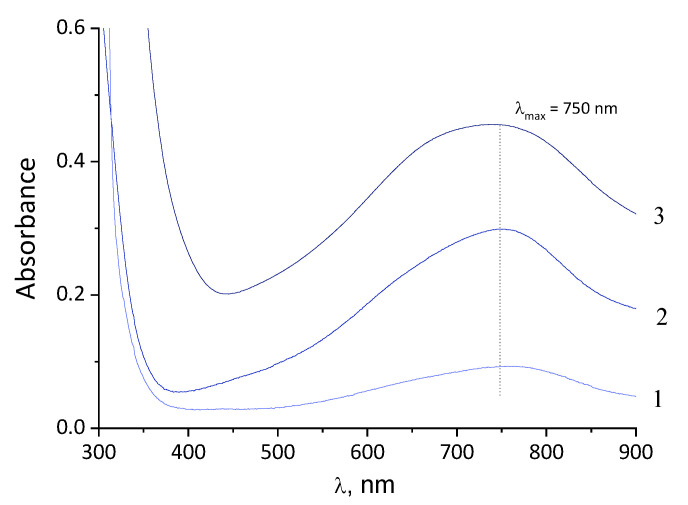
The UV/Vis spectrum of dispersion of molybdenum oxide clusters synthesized using various reducing agents: glucose (**1**), hydroquinone (**2**) and ascorbic acid (**3**) (the initial concentration of molybdate ion was constant).

The interaction of ammonium heptamolybdate with reducing agents in the above ratios leads to the formation of stable dispersions of molybdenum blue. The fact of their formation is confirmed by the appearance of an intense blue color and a change in the UV/Vis spectrum. In Figure 1 UV/Vis spectra are given for dispersions synthesized using glucose, hydroquinone and ascorbic acid. The spectra have an absorption maximum at 745 nm. This value of the absorption maximum is characteristic for toroidal molybdenum oxide nanoclusters belonging to the Mo_154−x_ family [33,36].

It should be noted that UV/Vis spectroscopy can also be used as a quantitative method for analyzing the dispersions of toroidal molybdenum oxide nanoclusters [12,33]. The change in the absorbance at the absorption maximum (λ_max_ = 745 nm) is directly proportional to the change in the concentration of nanoclusters in the dispersion. 

A kinetic experiment was carried out to establish and compare the rates of particle formation in the presence of various organic reducing agents. Absorbance was measured at the absorption maximum λ_max_ = 745 nm, which corresponds to the absorption maximum of toroidal molybdenum oxide nanoclusters [12]. Samples of molybdenum blue dispersions synthesized under optimal conditions (Section 4.3) were selected as the systems for the kinetic study.

**Figure 2:**
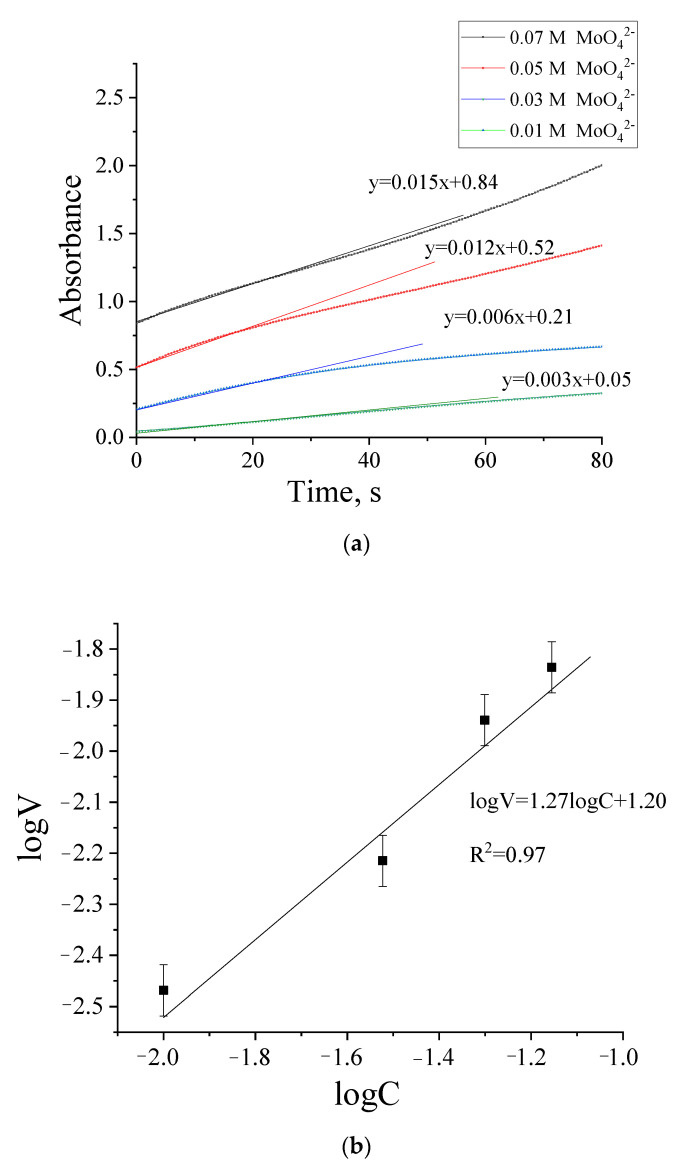
Time scan of the absorbance (λ_max_ = 745 nm) for dispersion of molybdenum blue, synthesized using ascorbic acid (**a**) and dependence of the rate of nanoparticles formation on concentration in logarithmic coordinates (**b**). (Dispersion was synthesized at molar ratios: (R/Mo) = 1.0; (H/Mo) = 0.8).

Figure 2 shows the time scans obtained at various concentrations of molybdate ions in the presence of ascorbic acid. As on can see the increase of the molybdate ions concentration, leads to the expected increase of particle formation rate.

The initial linear region of the kinetic plot was selected to estimate the formation rate. The results of the rate calculation are shown in Figure 2b in the coordinates of Equation (2) and were used to estimate the constants of that equation. Similar kinetic dependences and data were obtained for systems synthesized using hydroquinone and glucose at various concentrations of molybdate ions (see Table 1). 

As can be seen from the presented data, the rate and order of particle formation using ascorbic acid and hydroquinone are similar. In the case of glucose, the formation of particles occurs seven to eight times slower, the magnitude of the order of formation of nanoparticles changes.

Thus, the formation of nanoparticles of molybdenum blue in the presence of ascorbic acid and hydroquinone occurs much faster than in the presence of glucose, which may be due to the high reducing ability of hydroquinone and ascorbic acid. However, despite the fact that the rates of particle formation in the case of hydroquinone and ascorbic acid are similar, the highest concentration of particles, all other things being equal, is formed in the presence of ascorbic acid.

The low constant rate of self-assembly in the case of glucose can also be associated with a high concentration of glucose in the dispersion, which leads to an increase in the viscosity of the dispersion medium and, accordingly, to a decrease in the rate of self-assembly of molybdenum blue nanoparticles.

**Figure 3:**
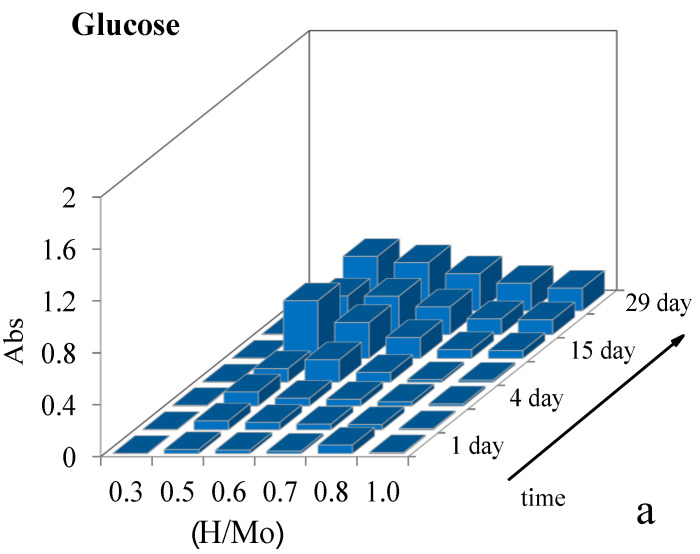

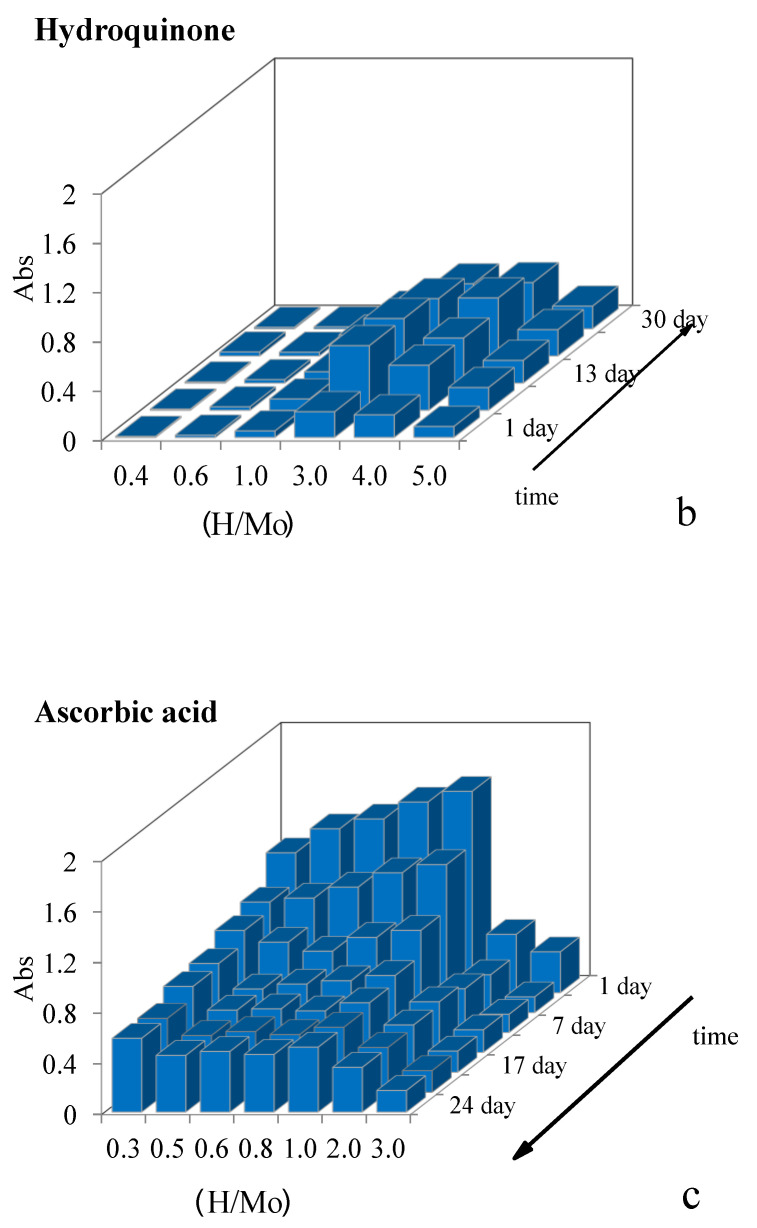
The dependence of the absorbance of samples of molybdenum blue on time and molar ratio H/Mo, synthesized by using glucose (R/Mo) = 7/1 (**a**), hydroquinone (R/Mo) = 4/1 (**b**) and ascorbic acid (R/Mo) = 1/1 (**c**).

It is known that the self-assembly of molybdenum-oxide clusters proceeds during time [8]. To establish the effect of the (H/Mo) molar ratio on the rate of the formation of molybdenum blue particles, series of samples were prepared with a constant (R/Mo) ratio and different acid contents. The absorbance at the wavelength corresponding to the absorption maximum (λ = 745 nm) was controlled during time. The time plots of the absorbance for various values of (H/Mo) are shown in Figure 3.

Over time, for systems prepared with glucose and hydroquinone, an increase in the absorbance and hence the number of particles of molybdenum blue is observed. The rate of their formation depends on the molar ratio of (H/Mo). There is a range of values in which the rate of formation is higher. For glucose this region is 0.5–0.8 and maximum of particles of molybdenum blue is observed at (H/Mo) = 0.5. At values of (H/Mo) less than 0.5 molybdenum blue formation does not occur. For hydroquinone this region is 1.0–5.0 and maximum of particles is observed at (H/Mo) = 1.5.

A different situation is observed in systems synthesized with ascorbic acid. Particle formation occurs on the first day in the entire range of the studied (H/Mo) ratios, that is probably due to the higher reducing capacity of ascorbic acid. The largest number of clusters is formed when the ratio (H/Mo) = 1. However, over time the absorbance decreases to a certain value, whereas the character of the spectrum does not change (the absorption maximum is 745 nm). Such behavior can be explained with a long time to establish equilibrium between the formed nanoclusters and the initial building blocks.

Despite the reducing agent used a constant value of absorbance of the synthesized dispersions is observed 21 days after synthesis. In contrast to small inorganic ions, which usually reach equilibrium state quickly giant molecules of polyoxometalates need time to reach an equilibrium state. The results obtained are in good agreement with the data available in the literature [19,27].

### 2.2. Dispersion and Nanoparticle Characterization

**Figure 4:**
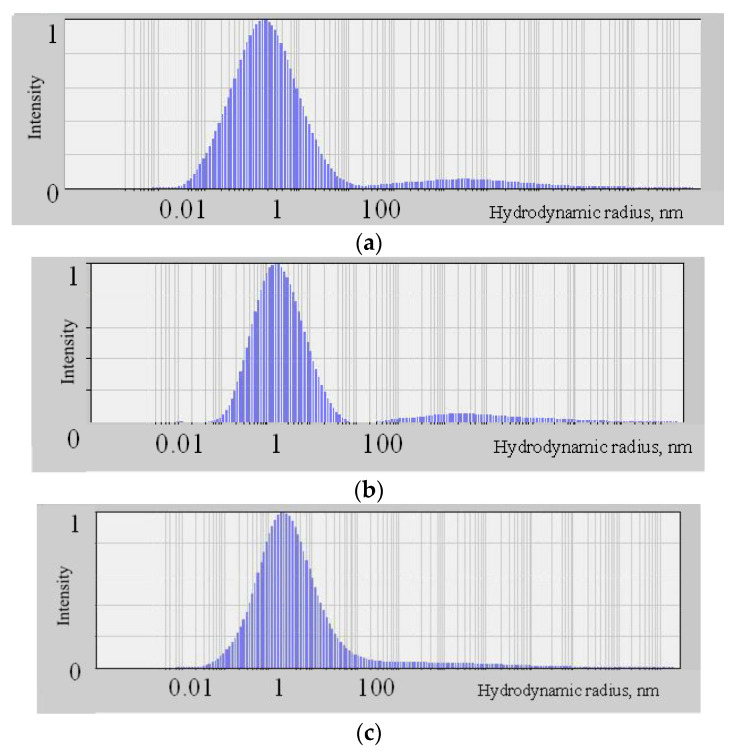
DLS distribution of molybdenum blue particles, synthesized by using glucose (**a**), hydroquinone (**b**) and ascorbic acid (**c**).

The particle-size distributions in molybdenum blues dispersions determined by DLS method are shown in Figure 4. We established, that the predominant hydrodynamic radius of the particles (R_h_ = 1.5 nm) did not change during the study period for all investigated samples (30 days).

In the Table 2 we summarized the optimized parameters for the synthesis with different reducing agents and how long it took to reach constant concentration of nanoclusters in dispersion and how long the concentration of particles is stable. The behavior of dispersions is different depending on the nature of the organic reducing agent. Dispersions synthesized using glucose and ascorbic acid can be stable for more than 2 and 1 months, respectively, so we suggested, that these organic reducing agents work not only as a reducing agent, but also as a stabilizer. Use of the hydroquinone as a reducing agent cannot provide the stability of molybdenum blue dispersion during long time. It should be also noted, that in the case of glucose and ascorbic acid is not necessary to add a significant quantity of hydrochloric acid, that is due to the acidic properties of these organic reducing agents.

**Figure 5:**
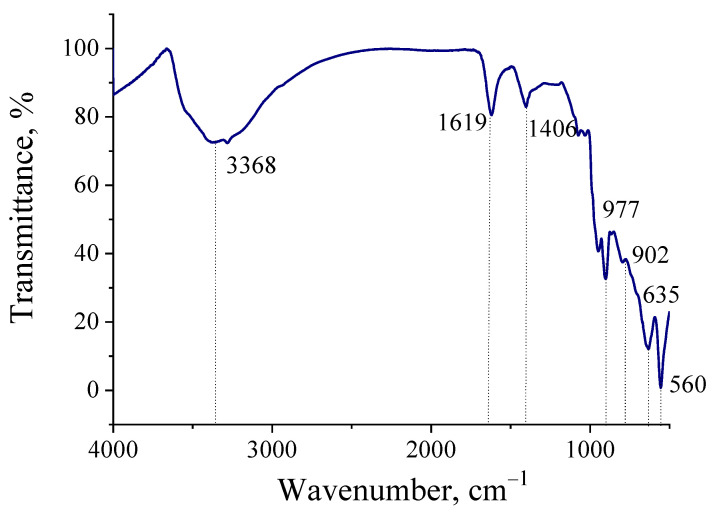
FTIR spectra of molybdenum oxide clusters isolated from dispersions synthesized by using glucose.

UV/Vis and FTIR spectroscopy is used to characterize molybdenum blue particles and its structure [21]. Figure 5 shows the UV/Vis spectra of molybdenum blue nanoparticles synthesized using various reducing agents. The particles were preliminarily isolated from dispersion by electrolyte coagulation (KCl), then the resulting precipitate was washed with water and ethanol to remove traces of organic substances (reducing agents and their oxidation products). Before measurements the samples of molybdenum oxide nanoclusters were placed in a non-polar solvent—ethanol.

The electronic absorption spectrum for all investigated samples contains a wide absorption band with maximum at 750 nm. The observed band, according to the literature [9], is characteristic for molybdenum blue which contain toroidal clusters (Mo_138_, Mo_150_, Mo_154_ and Mo_176_). Comparing the spectrum for the isolated particles with the spectrum of the initial dispersion, we concluded that after the separation of the particles, their structure does not change.

The typical IR spectrum for the molybdenum blue nanoparticles synthesized using glucose is shown in Figure 5. The IR-spectra for samples, synthesized using hydroquinone and ascorbic acid are very similar. The assignment of bands for molybdenum oxide nanoclusters is presented in Table 3.

Molybdenum blue particles have many hydrogen bonds ν (OH...H), as evidenced by the presence of a wide band in the region of 3100–3500 cm^−1^. These results are in good agreement with published data, which repeatedly noted a high content of OH groups on the surface of toroidal particles, as well as the presence of aqua ligands and intracluster water molecules (including the inner space of the toroidal cluster) [25]. The bands in the region of 1620 cm^−1^ correspond to deformation vibrations of water δH_2_O.

The region below 1000 cm^−1^ contains bands related to vibrations of the polyoxomolybdate framework, excepting band 1406 cm^−1^ corresponding to the δNH4^+^. In the range of 950–990 cm^−1^, closely spaced bands are observed, which are related to the Mo=O bond. A band of 957 cm^−1^ is also presented in this region, corresponding to stretching vibrations of the Mo–O–Mo bond [21]. The bands in the closer region of 490, 425 and 406 cm^−1^ refer to deformation vibrations of the δ (Mo–Oμ) bond, where oxygen is bonded to two or three molybdenum atoms.

The number of absorption bands observed on the spectra of the synthesized samples is in good agreement with the literature data and corresponds to the structure of the Mo_154_ toroidal clusters [3,25].

**Figure 6:**
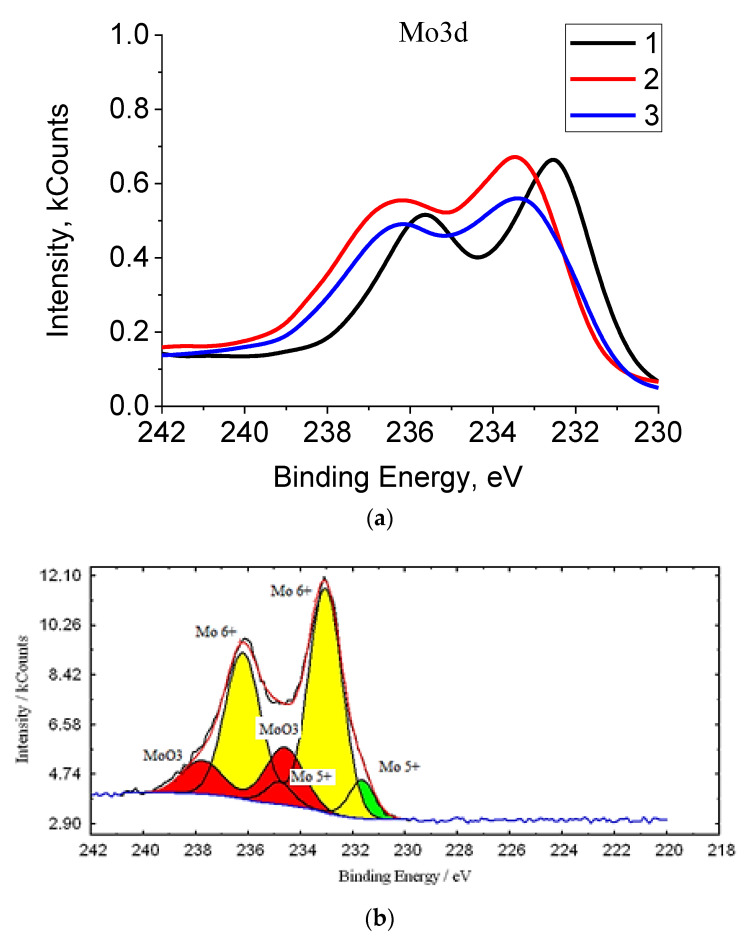
XPS spectrum: (**a**) Mo3d spectra of molybdenum oxide nanoclusters by using various reducing agents: glucose (**1**), hydroquinone (**2**) and ascorbic acid (**3**), and (**b**) typical spectra interpretation for sample synthesized using glucose.

To confirm the presence of reduced molybdenum Mo^V^ in the analyzed samples, XPS spectroscopy was used (Figure 6). According to the spectrum presented, the elemental composition of molybdenum blues is represented by molybdenum, oxygen and carbon, as well as the impurity content of potassium and chlorine (KCl was used to isolate particles from dispersions).

As can be seen, the spectrum of Mo3d electrons for samples of molybdenum blue synthesized using three different reducing agents differ from each other. Figure 6b shows the interpretation of this region of the spectrum.

For a more detailed consideration of the analysis results, the binding energies are presented in Table 4.

As can be seen from the data presented, the spectrum of Mo3d electrons for samples of molybdenum blue synthesized using glucose and hydroquinone is represented by three doublets: Mo^+6^ (electrons 3d5/2 and 3d3/2), Mo^+5^ (d5/2 and d3/2 electrons) and Mo^+6^ (MoO_3_) (electrons with binding energies 234.6 and 237.7 eV).

Molybdenum in molybdenum oxide clusters synthesized in the presence of ascorbic acid is presented in two forms: Mo^+6^ d5/2 and Mo^+6^ d3/2 and Mo^+5^ d5/2 and Mo^+5^ d3/2. Mo^+6^ in the form of MoO_3_ is absent or its amount is insignificant.

Thus, XPS spectroscopy confirms the presence of reduced molybdenum Mo^+5^ in the composition of molybdenum oxide clusters. The content of reduced molybdenum Mo^+5^ in molybdenum blues synthesized using glucose and hydroquinone is about 12%, with the use of ascorbic acid 29%. The results obtained agree with the literature data on the degree of reduction of molybdenum in toroidal clusters.

## 3. Discussion

Methods for the synthesis of aggregative stable molybdenum blue hydrosols using organic reducing agents (glucose, hydroquinone and ascorbic acid) have been developed. The synthesis conditions (R/Mo); (H/Mo) were established, which make it possible to obtain stable dispersions with reproducible properties, suitable for the further preparation of catalysts based on Mo_2_C [20,38,39,40].

From the results presented it follows that to obtain the maximum concentration of particles of molybdenum blue, the most effective reducing agent is ascorbic acid, the use of which allows the synthesis of dispersions even at a molar ratio of (R/Mo) up to 1.0. Therefore, the process of particle formation ends on the first day after synthesis. The stability of molybdenum blue dispersions maintains for no less than 1 month. Significant differences in the behavior of these systems are due to the more complicated mechanism of redox process with ascorbic acid [34]. So, stabilization of nanoparticles and the formation of an aggregative stable dispersion is possible only in a certain range of molar ratios (R/Mo) and (H/Mo), in contrast, for example, to glucose, which is a weaker reducing agent.

When using hydroquinone, a larger amount of reducing agent is required (R/Mo) = 4, the process of particle formation is completed 20 days after synthesis. Samples synthesized under optimal conditions are stable for 10 days. Hydroquinone acts only as a reducing agent and cannot provide the stability of dispersions.

The largest amount of reducing agent (R/Mo) = 7 is required to obtain molybdenum blue using glucose, which correlates with the lowest reducing ability of this compound under the selected synthesis conditions. The formation of particles occurs over a longer time (22 days).

Dispersions of molybdenum blue synthesized using glucose are the most stable. The results obtained also indicate that glucose and its oxidation products have a stabilizing effect on the dispersion of molybdenum blue, which is not observed when using hydroquinone.

Table 5 shows the advantages and disadvantages of using a certain type of reducing agent. The main advantage of glucose dispersions is their long-term stability. The use of hydroquinone makes it possible to obtain γ-MoC, which, according to the results of work [40], has a high catalytic activity in the dry reforming of methane reaction. Synthesis with the use of ascorbic acid makes it possible to obtain a dispersion with a high concentration of particles. The use of such dispersions as a precursor of molybdenum carbide makes it possible to vary the properties of the synthesized Mo_2_C [38].

## 4. Materials and Methods 

### 4.1. Materials

Molybdenum blue dispersions were synthesized at room temperature using the reagents ammonium heptamolybdate ((NH_4_)_6_Mo_7_O_24_∙4H_2_O, reagent grade), crystalline glucose (C_6_H_12_O_6_, reagent grade), hydroquinone (C_6_H_6_O_2_, reagent grade), ascorbic acid (C_6_H_8_O_2_, reagent grade) and hydrochloric acid (HCl, reagent grade). All reagents were delivered by CT Lantan (Moscow, Russia).

### 4.2. Synthesis of Molybdenum Blue Dispersions

Dispersions of molybdenum blue were synthesized via the reduction of molybdate solution by organic reducing agent. The synthesis was carried out at the constant molybdenum concentration 0.07 M at different molar ratios hydrochloric acid/molybdenum (H/Mo) and reducing agent/molybdenum (R/Mo), depending on the type of organic reducing agent. The system with glucose was synthesized at the constant molar ratio (R/Mo) = 7.0 and in the range (H/Mo) from 0.3 to 1.0. The systems with hydroquinone were synthesized at the constant molar ratio(R/Mo) = 4.0 and in the range (H/Mo) from 0.4 to 5.0. The systems with ascorbic acid were synthesized at the constant molar ratio (R/Mo) = 1.0 and in the range (H/Mo) from 0.3 to 3.0. 

### 4.3. Kinetic Study of Molybdenum Blue Nanoparticles Self-Assembly

The kinetic study of the nanoparticle self-assembly during the synthesis was conducted using UV/Vis spectroscopy. Samples of molybdenum blue dispersions, synthesized using glucose (R/Mo) = 7.0; (H/Mo) = 0.5; hydroquinone (R/Mo) = 4.0; (H/Mo) = 3.0 and ascorbic acid (R/Mo) = 1.0; (H/Mo) = 0.8, were prepared at the concentration of molybdate ion in the range 0.01–0.1 M.

The reagents (ammonium heptamolybdate solution, organic reducing agent and hydrochloric acid) were mixed in the quartz cell (volume—3 mL) directly before measurement. Time scan at the absorption maximum (λ_max_ = 745 nm) was taken for 100 s.

The rate of the process was described by the following equation [35]:

*V = kC^n^* (1)

where *V*—the rate of the reaction, *k*—the rate constant, *n*—the order of the reaction and *C*—the concentration of molybdate. The rate constant and the order of the reaction were determined from logarithmic form of the equation:

*logV = logk + nlogC* (2)

### 4.4. Characterization of Molybdenum Blue Dispersions

The pH value was measured by a HI-8314 pH/mV meter (Hanna Instruments, Vöhringen, Germany) with a combined electrode. UV/Vis spectra were recorded by Leki SS2110 UV scanning spectrophotometer (MEDIORA OY, Helsinki, Finland) using quartz cells.

The hydrodynamic radii of the particles in the molybdenum blue dispersions were determined via dynamic light scattering by Photocor Compact-Z analyzer (OOO Photocor, Moscow, Russia). The signal acquisition time was 30 min at a laser power of 20 mW and a wavelength of 658 nm.

FTIR spectra were measured by using Nicolet 380 IR Fourier spectrometer (Thermo Fisher Scientific Inc., Waltham, MA, USA) in compressed KBr pellets in the range from 350 to 4000 cm^−1^. The XPS spectra were recorded on ESCA + X-ray photoelectron spectrometer (OMICRON Nanotechnology GmbH, Taunusstein, Germany). The samples were exposed to X-rays (AlKα with excitation energy 1486.6 eV) under ultra-high vacuum conditions. The pass energy of the analyzer was 100 eV for registration of survey and 50 eV for measuring high-resolution spectra. The XPS peaks position were standardized by the C1s peak of hydrocarbon impurities from the atmosphere, the binding energy eV was taken 285.0 eV. The spectrometer is calibrated by the Au4f7/2 and Cu2p3/2 (the binding energy 84.0 and 932.6 eV, respectively). Decomposition XPS spectra into components after background subtraction was conducted according to the Shirley method. The peak position was determined with an accuracy of ±0.1 eV. For spectrum interpretation the software UniFit was used.

## 5. Conclusions

At first time a comparative analysis of the properties (rate constant, optimal synthesis conditions and time proceeding) of unique dispersed systems—molybdenum blues, synthesized using organic reducing agents of various nature, was done.

The influence of the (H/Mo) molar ratio on the rate of formation of molybdenum particles was established. For each reducing agent, the conditions (molar ratios of (H/Mo), pH value) for the formation of aggregative stable dispersion of nanoclusters with the maximum concentration of particles are determined. The kinetic experiment for dispersions obtained under optimal conditions made it possible to estimate the values of the rate constant and the order of formation of nanoparticles in the case of various organic reducing agents. It was shown that the rates of particle formation in the case of hydroquinone and ascorbic acid are close, while when glucose is used, the rate of formation of nanoclusters decreases by seven to eight times.

Based on the UV/Vis, FTIR, XPS spectroscopy and DLS data, it was shown that the particles of the dispersed phase are represented by nanocluster of a toroidal shape.

It was established, that hydroquinone acts exclusively as a reducing agent, ascorbic acid is a strong reducing agent, but in a certain range of molar ratios it can have a stabilizing effect, and glucose and its oxidation products are stabilizers.

## Figures and Tables

**Table 1:** The results of the kinetic study of the molybdenum blue nanoparticles formation (self-assembly).

Parameters	Reducing Agent		
	Glucose	Hydroquinone	Ascorbic Acid
Rate constant (k)	2.00	13.8	15.5
Reaction order (n)	0.35	1.16	1.19

**Table 2:** The synthesis conditions and time proceeding of molybdenum blue dispersions.

**Parameter**	**Reducing Agent**		
	**Glucose**	**Hydroquinone**	**Ascorbic Acid**
**Synthesis Conditions**			
Range (R/Mo)	5.0–9.0	3.0–6.0	0.6–5.0
Optimum (R/Mo)	7.0	4.0	0.8–1.0
Range (H/Mo)	0.5–0.8	1.0–4.0	0.5–1.0
Optimum (H/Mo)	0.5	3.0	0.8
pH	2.2	1.0	2.0
**Time Proceeding**			
Time to reach constant particle concentration, days	<20	<20	<10
Time of maintaining a constant concentration of particles, days	>60	<10	>30

**Table 3:** Assignment of several bands in the IR spectra of molybdenum oxide nanoclusters.

Band Position (cm^−1^)	Assignment	Reference Data
977 s 902 w	νMo=O	[37]
737 s 635 m	ν(Mo–μ_2_O–Mo) or ν(Mo–μ_3_O–Mo)	[37]
560 s	δ(O–Mo–O)	[37]
1619 s	δH_2_O	[37]
3368 s	ν(OH…H)	[37]
1406 w	δNH_4_^+^	[37]

s—strong, m—medium, w—weak, δ—bending vibrations, ν—stretching vibrations and μ_2_O/μ_3_O—bridged oxygen atom connected with two or three molybdenum, respectively.

**Table 4:** The results of XPS spectra of molybdenum clusters by using various reducing agents.

	Reducing Agent	Glucose	Hydroquinone	Ascorbic Acid
Doublet Name		Peak Position, eV		
Mo^+5^		231.66 234.81	231.9 235.05	232.22 235.37
Mo^+6^		233.05 236.20	233.23 236.38	233.50 236.66
MoO_3_ (Mo^+6^)		234.60 237.75	234.73 237.88	– –

**Table 5:** The comparative analysis of molybdenum blue dispersions synthesized using different organic reducing agent.

Reducing Agent	Glucose	Hydroquinone	Ascorbic Acid
Advantages	• long-term stability	• formation of γ-MoC (highly active catalyst of DRM *); • high formation rate	• high particle concentration; • high formation rate
Disadvantages	• low particle concentration; • low formation rate	• short-time stability	• short-time stability
Application	bulk and supported catalysts α-Mo_2_C/Al_2_O_3_	bulk catalysts α-Mo_2_C+ γ-MoC	bulk catalysts α-Mo_2_C+ ηMoC
Reference	[20,39,40]	[40]	[38]

* DRM—dry reforming of methane.

## Data Availability

The data presented in this study are available from the corresponding author upon request.

## Sample Availability

Samples of the compounds are not available from the authors.
